# Making the diagnosis of Chronic Fatigue Syndrome/Myalgic Encephalitis in primary care: a qualitative study

**DOI:** 10.1186/1471-2296-11-16

**Published:** 2010-02-23

**Authors:** Carolyn Chew-Graham, Christopher Dowrick, Alison Wearden, Victoria Richardson, Sarah Peters

**Affiliations:** 1 Primary Care Research Group, School of Community-Based Medicine, University of Manchester, Manchester, UK; 2 School of Population, Community and Behavioural Sciences, University of Liverpool, Liverpool, UK; 3 School of Psychological Sciences, University of Manchester, Manchester, UK

## Abstract

**Background:**

NICE guidelines emphasise the role of the primary care team in the management of patients with Chronic Fatigue Syndrome/Myalgic Encephalitis (CFS/ME). A key stage in effective management is making an accurate early diagnosis, supported by appropriate referral.

**Methods:**

A nested qualitative study within a multi-centre randomised controlled trial which aimed to explore GPs' views on their role in making the diagnosis of CFS/ME and subsequent management of patients in primary care. Semi-structured interviews with 22 GPs. Interviews were transcribed verbatim and an iterative approach used to develop themes from the dataset.

**Results:**

GPs described difficulties in defining CFS/ME and suggested that their role in making a diagnosis was to exclude physical causes for the patient's symptoms, but they reported little confidence in positively attributing the label of CFS/ME to a patient and their symptoms. GPs suggested that the label of CFS/ME could be potentially harmful for the patient. The role of referral to secondary care was debated and GPs struggled defining their own role in management of this group of patients.

**Conclusions:**

Until GPs feel comfortable making the diagnosis of CFS/ME and facilitating initial management, and have appropriate services to refer patients to, there will continue to be delays in confirming the diagnosis and patients presenting in primary care with fatigue may not receive appropriate care.

**Trial Registration:**

ISRCTN 74156610

## Background

Chronic Fatigue Syndrome (CFS) or Myalgic Encephalitis (ME) is a symptomatically defined condition with a principal complaint of severe, disabling fatigue which has been present for 6 months or more [1]. The symptom of fatigue must be of new or definite onset (i.e. not lifelong), and produces a substantial reduction in the patient's previous level of occupational, educational, social, or personal activities [2]. The diagnosis of CFS/ME remains controversial and debated but it is increasingly recognised as a clinical entity in primary care.

A recent report produced for the UK Chief Medical Officer by the CFS/ME working group [3], placed UK population prevalence at 0.2-0.4%, being twice as common in women as in men and affecting all social classes. The Chief Medical Officer report stated that patients should be diagnosed earlier and given better access to treatment, with a mutual management approach as its key component. This approach should be patient-centred therapy delivered by a trained multi-disciplinary team. The report also emphasised the need to provide these services locally [3]. This approach is echoed in the NICE guidelines for CFS/ME [4] which advocates a prominent role for primary care and suggests that healthcare professionals should aim to establish a supportive and collaborative relationship, working in partnership with the adult or child with CFS/ME, family, and carers to facilitate their effective management. The guidelines emphasise the importance of a definitive diagnosis and suggest that referral to a specialist should be made on the basis of the person's needs and symptoms: within six months of presentation to those with mild symptoms, within three to four months to those with moderate symptoms, and immediately to those with severe symptoms [4].

There is a literature on the negative views and scepticism that some GPs have expressed towards people with CFS/ME, [5,6] who, unsurprisingly therefore, are often dissatisfied with the medical care they receive [7]. Some doctors perceive patients with CFS/ME to have undesirable traits, and it has been argued that these negative attitudes towards patients often lead to problems with management [5]. The origins of these attitudes were perceived to be a lack of precise bodily location for the complaints, and the reclassification of the syndrome over time, producing conflict between doctor and patient over causation and management [8] and an apparent unwillingness of patients to disclose psychosocial components of their illness models [9]. Previous literature provides evidence of doctor-patient conflict due to disagreement over the causes and management of CFS [7,8], and of more positive attitudes amongst those GPs who accept CFS/ME as a recognisable clinical entity [10].

Surveys of GPs [10] and patients [11] quantify the difficulties patients with a CFS diagnosis and their GPs experience in their encounters [12]. Qualitative studies have explored these encounters and illustrate the difficulties found in managing patients with an established diagnosis [5,6,13]. There are no previous studies examining how the diagnosis of CFS/ME is made in primary care, the role played by the GP and the difficulties encountered in making that diagnosis.

The NICE guidelines encourage GPs to take an active role in the management of CFS/ME but, in particular, to make an early diagnosis. Recognising the condition and the impact it can have on the lives of patients and their families is a necessary preliminary to improving care [14]. It is thus essential to explore GPs' beliefs about the value of the label of CFS/ME, implications of the diagnosis and attitudes towards patients suffering with this condition. This was the aim of the study reported here.

## Methods

This was a qualitative study using semi-structured interviews with GPs in North West England working in practices that had been previously approached to participate in the FINE trial [15]. The study was reviewed and approved by the Eastern MREC (reference 03/5/62) and had PCT R&D approval. Sampling for the study was purposive and sought to achieve maximum variation in relation to GPs': age, ethnicity, practice location and size of practice as well as number of patients referred to the FINE trial at the time of the interview. Hence, we did not seek to recruit a representative sample of GPs, rather to access a range of views. Forty-six GPs in participating practices were invited by letter with an accompanying information sheet and contacted subsequently by telephone to discuss the study and ask them to participate in an interview. Of these, 22 agreed to be interviewed. Semi-structured interviews were conducted between 2005 and 2008. Interviews lasted between ten and 72 minutes (median duration 34 minutes).

An interview guide (see Appendix 1) provided a flexible framework for questioning and explored a number of areas: ideas about the cause of CFS/ME, previous experience of patients with CFS/ME, how the diagnosis of CFS/ME was achieved, and their role in management of these patients. The interviewer combined open questions to elicit free responses with focused questions for probing and prompting. Interviews were digitally recorded and transcribed verbatim.

Analysis proceeded in parallel with the interviews and was inductive, taking an interpretative stance [16,17]. Transcripts were read and discussed by researchers from different professional backgrounds (primary care (CCG, CD), psychology (SP, AW), and medicine (VR)) so increasing the trustworthiness of the analysis [18]. Coding was iterative and was informed by the accumulating data and continuing thematic analysis. Thematic categories were identified in initial interviews which were then tested or explored in subsequent interviews where disconfirmatory evidence was sought [16]. Interpretation and coding of data was undertaken by CCG, VR and SP individually and themes agreed through discussion within the whole team. The importance of reflexivity was discussed within the research team, with medical researchers (CCG, CD, VR) particularly reflecting on how their clinical perspective impacted on generation, analysis and interpretation of the data [17]. In reporting the final analysis the data are presented to illustrate the range and commonality of meaning of each category.

## Results

Data are presented verbatim and the GP identifier is displayed in brackets. Data are organised into five themes: defining CFS/ME, excluding the physical, potential harm from the label, the role of referral and moving on from making the diagnosis.

### Defining CFS/ME

GPs described a struggle, trying to make sense of a difficult set of symptoms and attributed different causes to the illness:

*'It's an illness they've got from life, whatever that is, and would be completely different for different people. So I wouldn't like to attribute it to one particular thing, but often it's to do with too much emotional stress going on in their life.' *(GP17)

*'I thought it was very much a sort of somatic presentation of a mental health problem and that was pretty much it. Probably quite patronising...sort of acknowledge that there was the fatigue, but didn't really see it as a separate entity. And I thought it was people sort of passively giving into symptoms and just sort of saying "right that's it", giving up. So I think it can be quite frustrating to work with.' *(GP9)

In addition, there was some debate over whether CFS/ME actually existed as a medical condition:

*'Well it's a relatively recent diagnostic term and I'm not so sure yet that it's recognised throughout the medical profession as an illness.' *(GP0)

*'I think that's increasingly wrongly medicalising it....to reinforce the fact that it's a medical disease that a specialist can cure, I think, gives the wrong messages.' *(GP16)

Such beliefs about CFS/ME necessarily will lead to difficulties in labelling the symptoms or making the diagnosis of CFS/ME.

### Excluding the physical

GPs articulated a process of diagnosis that prioritised excluding a physical cause for a patient's symptoms and presentation:

*'Well I'm a doctor, so I'd take a very full medical history, I would examine them and look for relevant things, and I'd do some investigations which would include looking for anaemia, thyroid problems, liver problems, vitamin D deficiency, that kind of thing' *(GP18)

GPs described looking for physical causes for symptoms and rarely suggested that exploring psycho-social issues would be important. Some GPs suggested that this bio-medical focus could be therapeutic since investigating symptoms communicates to the patient that the GP is interested in their symptoms and taking them seriously:

*'In the sense that if I sent, for example, the patient for a full blood count and ESR, the patient will feel two things, one is that he will feel that I have taken him seriously, if that comes back as negative, then they feel that "yes, the doctor has something to back up, to say I haven't got any cancer or anything", so they are doubly reassured. So I know it's a £10 investigation, but it is therapeutic in that respect...and they feel at least you have taken them seriously, and they go away reassured, that in itself is therapeutic, because the word therapy means to be cured doesn't it?' *(GP13)

Other GPs suggested that taking this bio-medical approach was important since they were excluding treatable causes of the patient's symptoms:

*'Clearly I'm excluding the treatable' *(GP14)

This implies that some GPs may feel that CFS/ME is not treatable, making their role in managing people whose symptoms are not easily categorized challenging.

### Potential harm from the label

Some GPs believed that the label of CFS/ME can be helpful for the patient in giving a name to their symptoms:

'*Some people like a label, some people like to know what's causing their symptoms whether it's the truth or not and some people are looking for a label to attach to their symptoms.' *(GP17)

However, this value was generally considered to be limited and short-lived:

*'At a superficial level it's empowering because it gives them control over their life and their work, but at a deeper level it prevents them from engaging fully with the existential conditions of their life which is what they can't cope with.' *(GP18)

Furthermore, the majority of GPs felt that the label of CFS/ME could be harmful because it did not offer a clear management pathway for either the GP or the patient:

*'I try to avoid it because once you give them the label you're actually setting them off on a track which will get them nowhere.' *(GP14)

*'Once you start labelling a patient if you're not careful you might have a self-fulfilling prophecy.' *(GP15)

### Role of referral

Those GPs who felt that making the diagnosis, or labelling the patient's condition, was helpful suggested that referring the patient to secondary care could potentially assist in achieving a diagnosis and providing support to GPs who lack confidence in making the diagnosis alone:

*'If someone else saw them who I felt was a good physician and also came to the same diagnosis as me then I would feel more confident that we were right.' *(GP15)

*'I'd be referring to them to actually make the diagnosis because I think they do extra testing. I think there may be other things in blood tests that they can search for that we don't.' *(GP22)

GPs, however, reported experiences of limited availability of potentially helpful places to support them in either making the diagnosis or managing the patient:

*'Well, I don't think there is anyone to refer to. The specialist clinic is a waste of time; they just hold their hands up, "what can we do? Why, what are they sending this to us for?"' *(GP14)

### Moving on from making the diagnosis

GPs alluded to the difficulties they had experienced working with patients with CFS/ME once the diagnosis was agreed:

*'I think you have to be very patient, accept yourself that you're not going to cure them but there are many things that you can do to alleviate the symptoms. Accept that it fluctuates, it's not something that comes on rapidly and then gradually gets better, it goes up and down. So you have to be prepared for a patient to appear to become worse despite your best efforts.' *(GP20)

The role of supporting the patient was stressed by respondents:

*'I think one of the crucial things for these kinds of people is for a doctor to say "I'm on your side, I'm going to be with you through thick and thin", and for the doctor to accept their relative powerlessness, but none the less to accompany the patient through this.' *(GP17)

A number of GPs reported frustrations with this work, implying that CFS/ME was difficult to manage as no "cure" was possible:

*'I mean, it's quite difficult for the Dr...So I think we have to learn, and you can walk alongside people with something you can't cure.' *(GP12)

And that the work invested in working with such patients is largely unrecognised:

*'That's terribly hard work for the doctor, it's very hard work. And there's no rewards in it...I don't refer the patient to twelve clinics so I've saved the health service millions of pounds and nobody gives a toss. Nobody says "thank you very much doctor, you're doing really hard work, you're seeing her every month and keeping her through a very difficult time, and you're going to do that year in year out for fifteen years." That kind of highly skilled work is completely disregarded and undervalued in the health service.' *(GP17)

## Discussion

### Summary of main findings

The study demonstrates a lack of confidence amongst GPs about making the diagnosis of CFS/ME and expressions of uncertainty about CFS/ME as a medical condition. GPs are reluctant to make the diagnosis of CFS/ME, possibly because for them no logical management decisions flow from the diagnosis. This contrasts with making the diagnosis of depression [19], perhaps because GPs believe they can offer treatment to most patients with depression which is more common compared with CFS/ME [8]. Some GPs expressed concern that giving the label of CFS/ME could actually cause harm to the patient and impair chances of recovery. In addition, the value of referral to secondary care was disputed, either in enabling a diagnosis to be made or offering support to the GP in supporting and managing the patient. This may be because of GPs' previous experience of the lack of specialist services but could also be because of their ambivalence about the role of the label of CFS/ME. GPs alluded to their own difficulties in making a diagnosis of a condition where they perceived no cure was possible and where their own role in supporting and managing patients with CFS/ME is under-valued.

### Strengths and Limitations

Data are presented from interviews with GPs over a wide geographical area and drawn from suburban, rural and inner city areas. This purposive sampling enabled us to access a range of views, intended to represent the *range *of views of the GP population from which the sample was recruited. However, because we employed a theoretical rather statistical sampling approach, the proportions of GPs holding the different views cannot be inferred. Further quantitative large scale surveying would be required, for example, to identify the specific percentages of GPs who consider providing a CFS/ME label to be helpful or harmful. Using authors from different professional and academic backgrounds is a recognised technique for increasing the trustworthiness of the analysis [18]. The opinions expressed in the interviews may not be similar to GPs in all practices, particularly those working in practices not participating in the FINE trial. GPs who participated in this trial, and the nested qualitative study, were likely to have been sensitized to CFS/ME and be more knowledgeable about the condition than the generality of their peers. Their uncertainties concerning diagnosis and management are, however, likely to be at least reflected, if not amplified, amongst other GPs.

### Comparison with existing literature

CFS/ME was seen as a contentious illness by many of the GPs in this study, viewing the condition of CFS/ME with scepticism, similar to descriptions in previous research [5,6], where GPs did not consider CFS/ME to be a genuine illness. This qualitative study confirms findings from previous quantitative work suggesting that GPs are unconfident in the diagnosis of CFS/ME [10].

Most GPs don't see enough patients with CFS/ME to become experienced in the diagnosis of this condition, and it has been shown that GPs can change their attitudes towards CFS/ME when a person who they know developed it [8]. The NICE guidelines suggest that negative attitudes and uncertainty about the condition make it difficult for GPs to establish a relationship with patients and that healthcare professionals should acknowledge the reality and impact of the condition and it's symptoms [4]. The NICE guidelines also recognise the need for a supportive and collaborative relationship with patients with CFS/ME to facilitate their effective management [4,14] and other guidelines stress that where possible CFS/ME should be managed locally [3]. GPs in this study reported lack of confidence in confirming the diagnosis of CFS/ME themselves, and that there were limited referral options locally. In addition, they expressed scepticism about the value of referral, having had previous negative experiences.

GPs in this study recognised that the diagnosis of CFS/ME is not the end-point for the professional or the patient and that managing a patient involves investment in a long-term relationship, which echoes previous work suggesting that diagnoses such as CFS/ME should be the beginning, not the end, of the therapeutic encounter [20]. Patients with CFS/ME are perhaps perceived to be more complex than those with other medically unexplained conditions, and GPs may feel less supported and competent to manage such patients [21,22]. Recognition of the complexity of this role is vital, and there is a need to acknowledge the valuable work that a GP might contribute to the management of patients with CFS/ME. For some conditions, treatment is straightforward and bio-medical, but for conditions like CFS/ME, GPs need to employ a wider range of skills, such as engagement, problem solving and developing a model of the illness in collaboration with the patient. GPs may lack confidence in taking a psychological approach with patients with CFS/ME [23] and commonly devalue their skills in psychological management of patients with unexplained symptoms [24].

### Implications for future research and clinical practice

Current training and education for GPs fails to prepare them for diagnosing and managing patients with CFS/ME as recommended by current guidelines. It seems that GPs do not have a clear model for CFS/ME. Educational initiatives are needed that are aimed at providing GPs with an acceptable model of the condition, supporting them in confidently making the diagnosis and helping them to initiate management based on their model. Passive educational interventions, however, have been shown to be ineffective [25] and more interactive educational initiatives perhaps involving patients as teachers should be considered [26].

## Conclusions

Until GPs feel comfortable making the diagnosis of CFS/ME and facilitating initial management, and have appropriate services to refer patients to, there will continue to be delays in confirming the diagnosis and patients presenting in primary care with fatigue may not receive appropriate care. It is necessary to recognise the complex nature of managing patients with long-term conditions. A model of support for GPs, which may include supervision [27], is needed. The provision of services to support GPs develop confidence to make the diagnosis of CFS/ME and manage and support patients with this complex debilitating condition [3,14] is necessary.

## Competing interests

The authors declare that they have no competing interests.

## Authors' contributions

CCG designed and managed this qualitative study. She contributed to the data collection and analysis and drafted the paper. She is guarantor for the study and paper. CD contributed to recruitment of GPs, data analysis and writing the paper. AW contributed to recruitment of GPs, data analysis and writing the paper. VR contributed to recruitment of GPs, data collection and analysis and writing the paper. SP designed and managed this qualitative study and contributed to the data collection and analysis and writing the paper. All authors read and approved the final manuscript.

## Appendix 1. The Interview Guide

*What follows is a guide; some themes may emerge spontaneously so the order of the questions varied as the interview and analysis phase developed allowing for the exploration of themes from the initial interview. The researcher probed and asked for examples to explore each topic as time permitted*.

1. Tell me about your experience of working with patients with chronic fatigue

a. What do you understand by the term CFS/ME?

b. What factors do you think cause it? Maintain it?

2. What do you do when someone presents with tiredness?

3. When and why would a diagnosis of CFS/ME come about?

a. How do you feel about making a diagnosis? Why?

b. What role do you think GPs have in diagnosing CFS/ME?

c. What role do guidelines (e.g. NICE) play in your diagnosis?

d. What could help with diagnosis?

4. What do you feel a diagnosis does for the patient? For how you manage the patient?

5. Do you refer patients to a specialist? If so, who to and why?

6. [How] does a diagnosis effect your interactions or relationship with a patient?

a. Do your perceptions/feelings about that patient change?

b. What kind of doctor-patient relationship helps manage effectively with these patients?

7. How would you approach a newly registered patient with a pre-existing diagnosis of CFS/ME?

## Pre-publication history

The pre-publication history for this paper can be accessed here:

http://www.biomedcentral.com/1471-2296/11/16/prepub
